# *In Vivo* Detection of Extrapancreatic Insulin Gene Expression in Diabetic Mice by Bioluminescence Imaging

**DOI:** 10.1371/journal.pone.0009397

**Published:** 2010-02-24

**Authors:** Xiaojuan Chen, Courtney S. Larson, Jason West, Xiaomin Zhang, Dixon B. Kaufman

**Affiliations:** 1 Department of Surgery, Feinberg School of Medicine, Northwestern University, Chicago, Illinois, United States of America; 2 Comprehensive Transplantation Center, Feinberg School of Medicine, Northwestern University, Chicago, Illinois, United States of America; University of Bremen, Germany

## Abstract

**Background:**

Extrapancreatic tissues such as liver may serve as potential sources of tissue for generating insulin-producing cells. The dynamics of insulin gene promoter activity in extrapancreatic tissues may be monitored *in vivo* by bioluminescence-imaging (BLI) of transgenic mice Tg(RIP-luc) expressing the firefly luciferase (luc) under a rat-insulin gene promoter (RIP).

**Methods:**

The Tg(RIP-luc) mice were made diabetic by a single injection of the pancreatic β-cell toxin streptozotocin. Control mice were treated with saline. Mice were subject to serum glucose measurement and bioluminescence imaging daily. On day eight of the treatment, mice were sacrificed and tissues harvested for quantitative luciferase activity measurement, luciferase protein cellular localization, and insulin gene expression analysis.

**Results:**

Streptozotocin-induced diabetic Tg(RIP-luc) mice demonstrated a dramatic decline in the BLI signal intensity in the pancreas and a concomitant progressive increase in the signal intensity in the liver. An average of 5.7 fold increase in the liver signal intensity was detected in the mice that were exposed to hyperglycemia for 8 days. *Ex vivo* quantitative assays demonstrated a 34-fold induction of the enzyme activity in the liver of streptozotocin-treated mice compared to that of the buffer-treated controls. Luciferase-positive cells with oval-cell-like morphology were detected by immunohistochemistry in the liver samples of diabetic mice, but not in that of non-treated control transgenic mice. Gene expression analyses of liver RNA confirmed an elevated expression of insulin genes in the liver tissue exposed to hyperglycemia.

**Conclusions:**

BLI is a sensitive method for monitoring insulin gene expression in extrapancreatic tissues *in vivo*. The BLI system may be used for *in vivo* screening of biological events or pharmacologic activators that have the potential of stimulating the generation of extrapancreatic insulin-producing cells.

## Introduction

The insulin gene is normally expressed in pancreatic β-cells through specific transcriptional control mechanisms [1], [2]. A highly conserved region approximately 400 bp immediately upstream of the transcription initiation start site confers both tissue-specific expression and metabolic regulation of the insulin gene [3]. Many transcription factors act upon this region forming a highly sophisticated transcriptional network that ensures precise regulation. Glucose is the major physiologic regulator of insulin gene expression; it coordinately controls the recruitment of transcription factors (e.g., pancreatic/duodenal homeobox-1, mammalian homologue of avian MafA/L-Maf, Beta2/Neuro D), the rate of transcription, and the stability of insulin mRNA [4].

Interestingly, it has been recently observed that hyperglycemia, with or without overt diabetes, activates insulin gene transcription and proinsulin production in multiple extrapancreatic tissues including liver, spleen, adipose tissue, thymus and bone marrow [5]. Hyperglycemia produced by glucose injections in mice led to the appearance of proinsulin- and insulin-positive cells in the liver within 3 days. Liver injuries such as those caused by chemical treatments [6], [7] were reported to induce activation and differentiation of hepatic oval cells to insulin-positive cells. Several lines of evidence [8], [9], [10], [11] also revealed that viral-vector mediated ectopic expression of pancreatic transcription and differentiation factors can induce liver cells to express insulin and cure diabetes in mice. Recently, it was reported [12] that adenoviral vector-mediated expression of transcription factor Neurogenin3 (NGN3) in liver resulted in a sustain expression of insulin in neo- islets cells derived most likely from the hepatic progenitor oval cells that secrete insulin in a glucose-responsive manner, stably reversing the hyperglycemia. The surprising capacity of the mature liver to serve as a potential source of tissue for generating functional insulin-producing cells provides a potential strategy for the treatment of diabetes.

In this study, we aim to establish an animal model to monitor insulin gene expression in extrapanceatic tissues *in vivo* and characterize the time-course of the insulin promoter activation in liver induced by hyperglycemia through bioluminescence imaging (BLI) [13], [14]. To do this, we induced hyperglycemia by streptozotocin (STZ) treatment in transgenic mice that express the firefly luciferase under the regulation of the 760 bp rat-insulin gene I-promoter. Rodents have two nonallelic insulin genes, insulin I and insulin II [15]. The insulin I gene is expressed in pancreatic beta cells and scattered cells in the thymus [16]. The insulin II gene, which is the ancestral gene and the homolog of the human gene, is expressed in pancreatic beta cells, and in the choroid plexus (secreting epithelium) region of the brain [17]. The expression pattern of luciferase in the transgenic mice used in this study was therefore expected to be similar to that of the endogenous insulin II gene. As a novel approach, BLI was used to monitor in real-time and to semi-quantify luciferase expression in the control and the STZ-treated diabetic mice *in vivo*. In addition, luciferase expression in the liver samples from the mice was examined by quantitative measurement of the enzyme activity *ex vivo*, and by immunohistochemistry to identify cells expressing the enzyme. To confirm that the transgene expression mimics that of the endogenous insulin gene, insulin gene expression in the liver was examined by semiquantitative Reverse Transcription (RT)-PCR analyses in autopsy liver samples.

## Materials and Methods

### Mice

A transgenic mouse line, FVB/N-Tg(RIP-luc) containing the firefly luciferase gene under the regulation of the rat insulin promoter II (RIP, 760 bp) that specifically and constitutively expresses firefly luciferase in the pancreatic islet β-cells (Caliper Life Sciences, Alameda, CA), was used for both experimental and control groups. All mice were housed in a barrier facility at Northwestern University and were used at ages 3-4 months. All animal procedures were approved by the Animal Care and Use Committee at Northwestern University.

### Diabetes Induction

FVB/N-Tg(RIP-luc) mice were made diabetic by a single intraperitoneal (i.p.) injection of the pancreatic β-cell toxin streptozotocin (STZ, 220 mg/kg body weight dissolved in saline, Sigma-Aldrich, St. Louis, MO). Serum glucose levels were measured daily using a glucometer (One Touch Basic, Lifescan Inc., Milpitas, CA). Mice that demonstrated serum glucose levels greater than 300 mg/dL on two consecutive days were considered diabetic. Control mice received an i.p. injection of saline solution.

### In Vivo Bioluminescence Imaging

In preparation for BLI, control and diabetic FVB/N-Tg(RIP-luc) mice were anesthetized with 2.5% isofluorane in air. Substrate luciferin potassium salt (Molecular Therapeutics Inc., Ann Arbor, MI) dissolved in PBS (40 mg/ml) was administered via a single i.p. injection at a dose of 200 mg/kg body weight. The mice were placed in the camera chamber, where a controlled flow of 2.0% isofluorane in air was administered through a nosecone via a gas anesthesia system designed to work in conjunction with the BLI system (IVIS® 200, Caliper Life Sciences). Mice were imaged for one-minute durations on both the dorsal and ventral sides at medium-resolution with a field of view of 20 cm at 10 minutes post luciferin injection. A gray-scale body image was collected and overlaid by a pseudo-color image representing the spatial distribution of detected photons. The BLI signal intensities emitted from the liver region of the mice were quantified using a fixed region of interest (ROI) of 2×1.5 cm in size from dorsal images and expressed as photons per second of light. Background images were collected daily and background subtractions were automatically calculated by the Living Image software. Ventral images were collected solely for visual purposes.

### Quantitative Luciferase and Total Protein Measurements

Autopsy mouse liver tissue samples were collected from either control normal glycemic mice or mice that had been treated with STZ and exposed to hyperglycemia for 8 days. The randomly collected liver samples (∼5 mm^2^) were placed in 400 µl of Passive Lysis Buffer (Promega Corp, Madison, WI) and were homogenized using a handheld tissue homogenizer (Pellet Pestle® Motor, Kontes). Tissue homogenates were centrifuged and the supernatant were used for luciferase activity and protein concentration assays. For the luciferase activity measurement, the *In Vitro* Luciferase Assay System (Promega) was used according to instructions provided by the manufacturer. Tissue homogenates (20 µl) and the luciferase substrate (100 µl) were mixed and the luminescence generated from the reaction was measured using a Sirius Luminomter (Berthold Detection Systems). Background luminescent readings were obtained and were subtracted from the luminescent data. Protein concentrations were determined using the BCA Protein Assay Kit (Pierce, Rockford, IL) according to the manufacturer's protocol and analyzed using a Coultar® UV Max microplate reader and the associated Softmax Pro version 3.1.2 software (Molecular Devices, Sunnyvale, CA). The luminescence of each of the sample lysates was calculated and expressed as relative light units (RLU) per second per microgram of protein.

### Tissue Preparation, Antibodies, and Immunohistochemical Analysis

Autopsy mouse pancreas and liver tissue samples were collected from Tg(RIP-luc) mice that were either control normoglycemic or that had been treated with STZ and exposed to hyperglycemia for 8 days. The samples were fixed in 10% buffered-formalin for 48 hours prior to paraffin embedding. Tissue sections of 3∼4 microns were cut and placed on charged slides, allowed to air dry before placing in a 60°C oven for at least 1 hour for adhesion. After deparaffinization and antigen retrieval, the tissue sections were incubated with primary rabbit polyclonal antibody to Firefly Luciferase (Abcam Inc, Cat#ab21176, 1∶500) for 30 min at room temperature, followed by incubation with a ready-to-use EnVision™+ Single Reagents (Dako, Cat#K4003) for 15 min at room temperature. Images of the graft sections were acquired using a Zeiss fluorescence axial microscope attached with a digital camera.

### RNA Extraction and Analysis of Tissue mRNA Expression

Total RNA was prepared from frozen liver samples using TRI Reagent (Molecular Research Center) with bromochloropropane according to the manufacturer's protocol. RNA quantity and quality was determined spectrophotometrically and 0.7 µg total RNA was used for reverse transcription in the presence of MMLV-Reverse Transcriptase and random hexanucleotides (Promega). The reverse-transcribed samples were denatured and amplified by PCR in the presence of GoTaq Flexi DNA Polymerase (Promega) in a PCR System 6700 (Applied Biosystems) for over 30 cycles (94°C, 45 sec; 58.5°C, 45 sec; 72°C, 45 sec) with a final extension time (72°C, 4 min). A sham reaction without the reverse transcriptase was performed in order to ensure that PCR amplification did not arise from genomic DNA contamination. PCR primers used for the detection of insulin-I were: forward 5′-ccatcagcaagcaggtca-3′; reverse 5′-ccacacaccaggtagagagc-3′; insulin-II: forward 5′-cctgctggccctgctctt-3′ and reverse 5′-ggctgggtagtggtgggtcta-3′. The PCR products were separated by electrophoresis in 1.8% agarose stained with ethidium bromide. All reaction templates were normalized using the same PCR conditions with GAPDH expression using primers: forward 5′-accacagtccatgccatcac-3′ and reverse 5′-tccaccaccctgttgctgta-3′.

### Data Analysis

The mean ± standard error (SE) of the BLI signal intensity and luminescence for both groups were calculated and the unpaired two-tailed Student's t-test was used for statistical analysis. A probability (*p*) value of <0.05 was considered to be statistically significant.

## Results

### In Vivo Bioluminescence Imaging and Luciferase Expression

**Figure 1:**
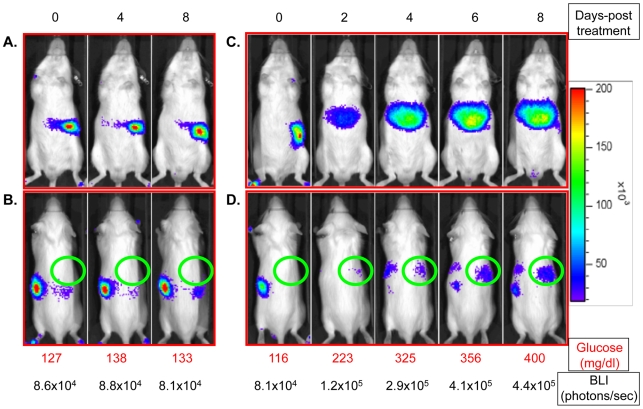
Representative mouse bioluminescence images. Panels A and B are ventral and dorsal bioluminescence images of a representative control mouse on days 0, 4 and 8 after the start of the experiment. Panels C and D are ventral and dorsal bioluminescence images of a representative mouse on days 0, 2, 4, 6 and 8 after treatment of STZ. Images were standardized to a light intensity scale on the right. The daily mouse blood glucose level and the liver ROI luminescent intensity were recorded under each image.

Mouse serum glucose levels and luciferase expression in the liver were evaluated daily for 8-10 days following treatment with saline solution (Control, n = 6) or STZ (n = 6). Figures 1A and 1B show the ventral and dorsal bioluminescence images of a representative control mouse taken on days 0, 4 and 8 after the start of the experiment. The blood glucose reading and the BLI signal intensity of the circular area (ROI, the liver region of interest) were recorded under each image. As expected, strong and consistent levels of BLI signals were detected from the pancreas region on all days tested as the serum blood glucose level stabled. The strongest signal was detected from the tail-portion of the pancreas while the head of the pancreas can be more clearly visualized from the dorsal side. The BLI signal intensity from the liver ROI was near the background reading (8.0∼9.0×10^4^ photons/second) at all times tested.

In comparison, Figures 1C and 1D represent the luminescence imaging of a STZ-treated FVB/N-Tg(RIP-luc) mouse in ventral and dorsal positions on days 0, 2, 4, 6, and 8 as serum blood glucose levels increased over a period of eight days. As demonstrated by Figure 1C, there was a dramatic decrease in pancreatic luminescent signal soon after the STZ treatment as the β-cell function was lost, and a concomitant spatial shift of the luminescent intensity to the area where liver is located as serum blood glucose levels rose above 300 mg/dl. The location where the luminescence was detected was very similar to that of luminescence emitted from non-transgenic FVB/NJ mice transplanted with the luciferase-tagged islets in the liver [13]. The shift of luminescent expression also occurred on the dorsal side of the mouse as shown in Figure 1D. To quantify the changes in bioluminescence intensity, a fixed circle (ROI) was placed over the area of the upper right abdomen on the dorsal side as shown in the figures. This specific area of the liver was targeted because of its distant proximity from the pancreas, thus minimizing pancreatic signal interference. As shown in Figure 1D, the BLI signal obtained from the liver ROI of a representative mouse on day 0 was 8.1×10^4^ photons/second, similar to that of the controls. The signal intensity increased 5.43 fold to 4.4×10^5^ photons/second over the course of eight days under hyperglycemia. Enhanced luminescence was detected by BLI in the liver region as early as 24 hours following treatment with STZ when the blood glucose level rose near or above 200 mg/dl (data not shown).

We also observed enhanced luminescence signal on the left side of the mouse (Figure 1D) with the progression of hyperglycemia. The location and the shape of the BLI images suggested that it was emitted from the spleen. The spleen is one of the organs that demonstrated positive insulin gene expression under diabetic conditions [5]. In addition, some of the STZ-treated mice showed an enhanced luminescence signal from the brain area as detected by BLI (data not shown). As discussed above, brain tissue is known to be an active site for insulin II gene.

**Figure 2:**
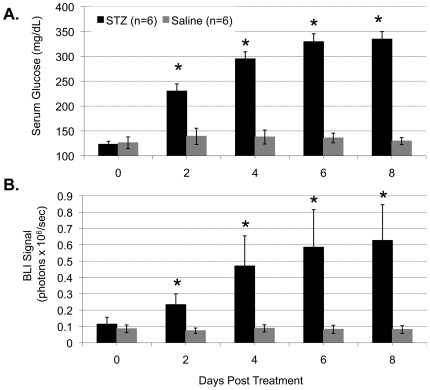
Quantified average mouse blood glucose level and BLI signal intensity from the mouse liver region. Panels A and B are average measurements of, respectively, non-fasting blood glucose and BLI signal intensity from the liver ROI of the control saline-treated mice (n = 6) and diabetic STZ-treated mice (n = 6). “*” indicates a significant difference between the measurements of the STZ-treated mice and that of the control mice on the same time point.

Figure 2A summarizes the average serum glucose levels from both the saline- and the STZ-treated mice on days 2, 4, 6, and 8 following treatment. As shown, the average serum glucose levels on day 0 for the control and treatment groups were 126±11.7 and 123±15.4 mg/dl respectively (*p* = 0.81). While the saline-treated control mice maintained normoglycemia during the entire study period, the STZ-treated mice had a progressive increase in the blood glucose level each day thereafter (230±14, 295±14, 329±16, and 334±15 mg/dl, respectively), resulting in a significant difference from that of the control mice on days 2, 4, 6 and 8 (*p* = 0.002, 2.98×10^−5^, 4.49×10^−6^ and 1.25×10^−6^ respectively). Moreover, the average serum glucose level of the STZ-treated mice on day 0 before treatment was significantly different from that of days 2, 4, 6 and 8 (*p*<0.001 for all time points).

With the increase in the blood glucose level, there was a significant increase in luciferase expression in the liver of STZ-treated mice. Figure 2B represents the quantified average BLI signal intensity from the mouse liver region on days 0, 2, 4, 6, and 8 following treatment with saline or STZ. The BLI signal of the liver was quantified from the fixed ROI on the dorsal image as demonstrated in Figures 1B and 1D. The mean (± SE) BLI intensities on day 0 for the control and treated mice were 8.56±0.02×10^4^ and 11.10±0.04×10^4^ photons/second respectively. No statistical differences between the groups were found (*p* = 0.55). The signal intensity from the liver area of STZ-treated mice increased significantly each day thereafter (23.30±0.66×10^4^, 47.0±1.85×10^4^, 58.5±2.30×10^4^ and 62.60±2.19×10^4^ photons/second respectively), resulting in a statistical difference from the control mice on days 2, 4, 6, and 8 (*p* = 0.044, 0.046, 0.012, and 0.031 respectively). An average of 5.64 fold increase in the liver signal intensity was detected in the mice that were exposed to hyperglycemia for 8 days. The average BLI of the STZ-treated mice on day 0 before treatment was statistically different from days 2, 4, 6, and 8 (*p* = 0.03, 0.0003, 0.0001, and 0.0002 respectively), whereas no significant differences were observed in the control mice.

### Ex Vivo Luciferase Enzyme Activity Measurement

**Figure 3:**
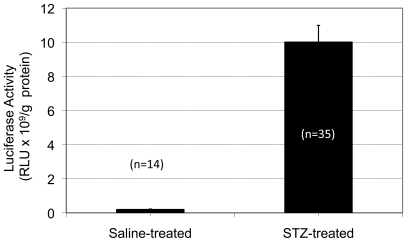
Quantified liver luciferase enzyme activity *ex vivo*. The firefly luciferase enzyme activity was measured in the liver samples from the control (n = 14) and the STZ-treated (n = 35) mice. The enzyme activity is expressed as the relative light unit (RLU) detected per gram of protein.

To confirm the *in vivo* BLI finding that luciferase expression under the insulin promoter was elevated in the liver under hyperglycemia, liver autopsy samples were obtained on day 8 and luciferase assays were performed on tissue lysates from the liver to quantify the luciferase expression in control and treated mice. A total of 14 random control samples and 35 random STZ-samples from 6 mice in each group were analyzed. Figure 3 represents the mean (± SE) luciferase activity expressed in RLU per gram of protein. As shown by this figure, the mean luciferase activity for the control liver was 0.30±0.03×10^9^ whereas the luciferase activity for the hyperglycemia-liver produced an average of 10.00±0.10×10^9^. There was a 34-fold induction in the luciferase activity in the liver after an eight-day exposure to hyperglycemia with a very large statistical difference (*p* = 6.76×10^−10^) compared to that of the controls. Luciferase activity in the brain tissue homogenate was also measured, quantified and shown to be 2-fold higher in the STZ-treated mice than that of the controls (data not shown).

### Immunohistochemical Staining of Cells Expressing Luciferase

**Figure 4:**
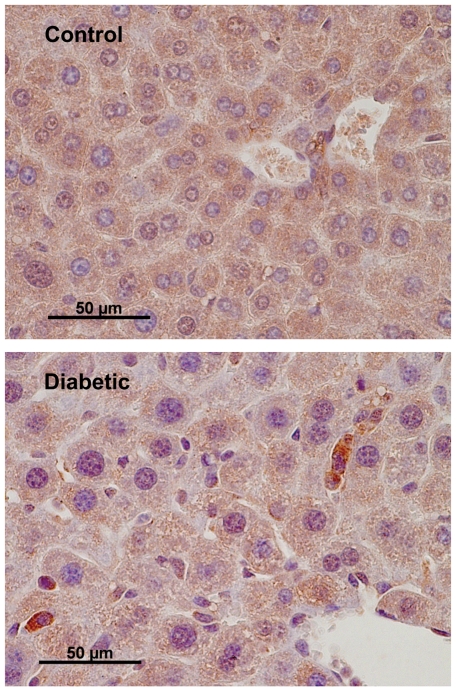
Representative images of mouse liver sections stained for luciferase. Control and diabetic Tg(RIP-luc) mouse liver samples were stained by the immunoperoxidase technique for firefly luciferase. Scales represent the original magnification.

For the identification of cells expressing luciferase, liver tissue samples from the Tg(RIP-luc) mice, both diabetic and non-treated, were subject to immunohistochemical staining with an anti-luciferse antibody. As shown in Figure 4, luciferase-positive cells were detected in the liver samples from the STZ-induced diabetic mice, but not in the liver samples of the non-treated control transgenic mice. These cells are likely hepatic oval cells since they are small in size (approximately 10 µm), with a large nucleus-to-cytoplasm ratio and an oval-shaped nucleus, and are located in the periportal region of the liver. No hepatocytes were detected positive for luciferase expression. In the pancreas samples of the Tg(RIP-luc) mice, luciferase-positive cells were only detected in islets (data not shown).

### Liver Insulin Gene Expression Analyses

**Figure 5:**
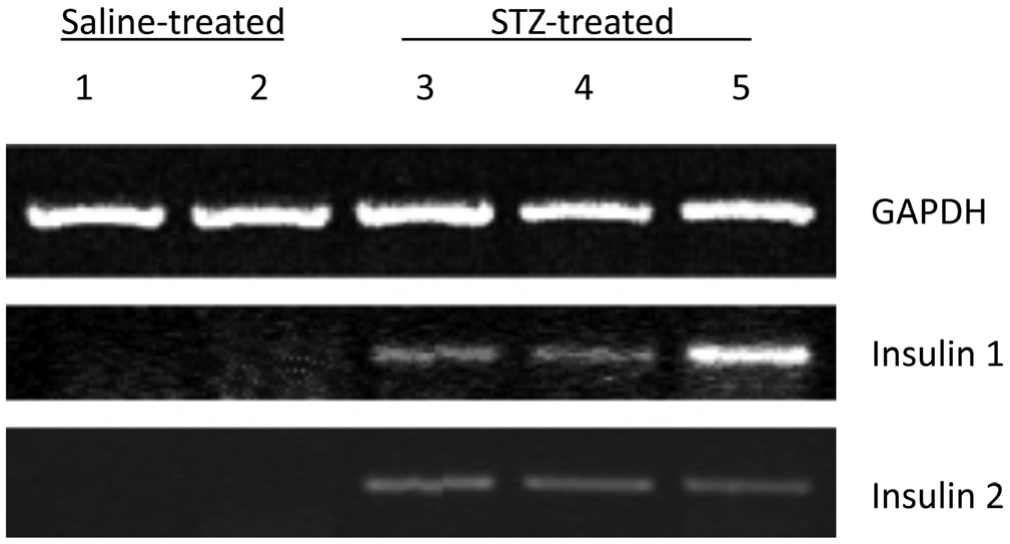
Semi-quantitative RT-PCR detection of insulin gene expression in mouse liver. Liver samples 1 and 2 were from control mice; samples 3, 4, and 5 were from mice exposed to hyperglycemia for eight days. Total RNA was subject to RT-PCR detection of insulin-I and insulin-II transcripts. A fragment of mouse GAPDH cDNA was amplified for internal control. The amplified products were separated on a 1.8% agarose gel and visualized by ethidium bromide staining.

To determine if the transgene expression detected by BLI was associated with the endogenous insulin gene promoter activity, the expression of insulin-I and insulin-II were examined by semiquantitative RT-PCR analysis of RNA samples prepared from the liver samples of control and STZ-treated mice. As shown in Figure 5, the expression of both the endogenous insulin-I and insulin-II genes were elevated in the liver RNA samples from animals exposed to hyperglycemia for 8 days (samples 3, 4, and 5). No insulin transcripts were detected in the control liver samples (samples 1 and 2).

## Discussion

With Type I diabetes, and in some patients with Type II diabetes, the lack of insulin can best be supplemented by providing new insulin-producing cells. Potential alternative approaches for obtaining more insulin producing cells for the treatment of diabetes include proliferation of existing β-cells, differentiation from adult endocrine progenitor cells or embryonic stem cells, and reprogramming of non-β-cells such as acinar or liver cells to β-cells [18]. Each of these approaches has distinct merits and limitations, and are at the early stages of investigation and development.

As the above mentioned approaches of obtaining more insulin-producing cells from β- or non-β-cell origins are being investigated, the ability to observe, locate and quantify the cells producing insulin over time *in vivo* in real time, has become increasingly important. In this study, we sought to validate the use of a non-invasive *in vivo* imaging system for the detection of induction of non islet-β cells such as liver cells to insulin-producing cells. The induction model used in this study has been reported previously [5], [6], [7]. The novelty of our investigation lies in the application of BLI to the model in which the transgenic mice expressing the reporter gene coding for the firefly-luciferase driven by a rat insulin gene promoter were treated with STZ to induce hyperglycemia, a condition known to induce the insulin gene expression in liver cells. We demonstrated that BLI allows the detection of activation of insulin gene *in vivo* via reporter luciferase expression in a timely and semi-quantitative manner. In addition, the activation site (organ) can be visualized and identified.

Non-invasive *in vivo* imaging can provide real-time spatial and temporal information about the location, function, and viability of cells of interest in intact living animals. The luciferase reporter system has been used for noninvasive measurements of gene expression in animals. This molecular imaging strategy has been effectively incorporated into studies of small animal models of human biology and disease [19], [20]. The tool offers the advantage of noninvasive *in vivo* assessment of the molecular and cellular events that are often targets of therapy; as such, these events can be studied in individual animals over time. This reduces the number of animals required for a given study and improves the quality of the data set, as the temporal measurement allows for each animal to serve as its own control. As have been reported in our previous studies using BLI to monitor islets after transplantation, BLI is a sensitive method for monitoring gene expression in minimal amounts of cells as it is capable of detecting as few as 10 luciferase-expressing islets transplanted to the liver via portal vein infusion [14]. Nonetheless, the measurement of luciferase activity *in vivo* is challenged by the fact that light transmission is subjected to attenuation by tissues overlaying the light source in live animals. In this study, we observed luminescence from liver, brain (not shown), and possibly spleen of mice under hyperglycemia. This does not exclude the possibility that the insulin genes in other tissues such as adipose and bone marrow are also activated, as reported by others [5]. Rather, it suggests that the level of insulin gene expression in these other tissues may be, if present, lower than that in the liver and beyond the detection limit of the imaging modality. It has been reported that hepatic oval cells, the liver progenitor cells, can be activated and differentiated toward pancreatic beta-cell phenotype under conditions such as high concentrations of glucose, liver injury (e.g. STZ-treatment) or NGN3 overexpression [6], [7], [12], [21]. Consistent with these reports, the immunohistochemical staining results in this study showed that, under the experimental condition, luciferase expression driven by the rat insulin promoter was mainly activated in cells with oval cell morphology.

In conclusion, BLI is a valid, sensitive, and semi-quantitative technology for monitoring insulin gene expression when used in combination with transgenic mice expressing reporter gene luciferase under the regulation of the insulin gene promoter. It may be used for *in vivo* screening of pharmaceutical drugs that have the potential of stimulating the generation of new sources of insulin-producing cells in aspects previously inaccessible to investigation.
